# Temporal Trends in Loneliness Across Four Decades in the Framingham Heart Study

**DOI:** 10.1093/geroni/igaf122.4019

**Published:** 2025-12-31

**Authors:** Allison Mays, Matthew Scott, Alexa Beiser, Mitzi Gonzales, Joel Salinas

**Affiliations:** Cedars Sinai Medical Center, Beverly Hills, California, United States; Boston University, Boston, Massachusetts, United States; Boston University, Boston, Massachusetts, United States; Cedars Sinai Medical Center, Beverly Hills, California, United States; New York University Grossman School of Medicine, New York, New York, United States

## Abstract

Using the Framingham Heart Study, we aimed to describe the prevalence of loneliness within age groups across four decades. Participants completed the Center for Epidemiologic Studies Depression Scale (CES-D Scale) and responded how often they felt lonely in the past week. We included in our analysis 30,186 observations longitudinally collected between 1980-2019. Loneliness reported at least once weekly was more prevalent in younger age groups, decreased in mid-life and then increased in older age in a U shaped curve as in prior literature; this trajectory persisted across decades with total prevalences of: 26.6%, 22.5%, 20.6%, 18.3%, 16.5%, 23.1%, and 33.7% in age groups 25-34, 35-44, 45-54, 55-64, 65-74, 75-84 and 85-94 respectively. Loneliness three or more times per week decreased from the 1980s for all ages with continued declines in younger (25-34; 35-44) and older (75-84; 85-94) age groups. Despite these decreases, loneliness prevalence remained highest in the oldest age groups (75-84; 85-94) which reported rates of 11.6% and 15.9% respectively, compared to 5.7-6.9% in younger participants. Loneliness prevalence in ages 75-84 and 85-94 declined across decades with rates respectively of 22.4% and 36.0% from 1980-1989; 11.4% and 15.3% from 1990-1999; 9.7% and 13.9% from 2000-2009; and 7.0% and 9.8% from 2010-2019. However, prevalence rates across ages still maintained a J shaped curve. Loneliness has decreased from the 1980s in the Framingham Heart Study, though older adults ages 75-84 and 85-94 maintained the highest prevalence of loneliness. Further research into factors contributing to declining rates of loneliness is merited.

